# Real-world effectiveness of antidepressant use in persons with schizophrenia: within-individual study of 61,889 subjects

**DOI:** 10.1038/s41537-023-00364-x

**Published:** 2023-05-26

**Authors:** Arto Puranen, Marjaana Koponen, Markku Lähteenvuo, Antti Tanskanen, Jari Tiihonen, Heidi Taipale

**Affiliations:** 1 Department of Forensic Psychiatry, Niuvanniemi Hospital, University of Eastern Finland, Kuopio, Finland; 2 School of Pharmacy, University of Eastern Finland, Kuopio, Finland; 3 Centre for Medicine Use and Safety, Faculty of Pharmacy and Pharmaceutical Sciences, Monash University, Parkville, VIC Australia; 4 Department of Clinical Neuroscience, Karolinska Institutet, Stockholm, Sweden; 5 Center for Psychiatry Research, Stockholm City Council, Stockholm, Sweden

**Keywords:** Schizophrenia, Psychosis

## Abstract

The aim of this study was to investigate the real-world effectiveness of antidepressant use in persons with schizophrenia. The register-based study cohort included all 61,889 persons treated in inpatient care due to schizophrenia during 1972–2014 in Finland. The main outcome was hospitalization due to psychosis and secondary outcomes included non-psychiatric hospitalization and all-cause mortality. We used within-individual design to compare the risk of hospitalization-based outcomes during the time periods of antidepressant use to antidepressant non-use periods within the same person, and traditional between-individual Cox models for mortality. The risk of psychosis hospitalization was lower during antidepressant use as compared to non-use (adjusted Hazard Ratio, aHR, 0.93, 95% CI 0.92–0.95). Antidepressants were associated with a decreased risk of mortality (aHR 0.80, 95% CI 0.76–0.85) and a slightly increased risk of non-psychiatric hospitalization (aHR 1.03, 95% CI 1.01–1.06). In conclusion, these results indicate that antidepressants might be useful and relatively safe to use in this population.

## Introduction

Depression is common among persons with schizophrenia, reaching a prevalence of 33% according to a recent meta-analysis^1^. Lack of treatment or treatment response to depression may result in serious consequences among persons with schizophrenia, including alcohol misuse and more frequent paranoid delusions^2^. Consequently, antidepressants are widely used in persons with schizophrenia to treat a variety of symptoms, such as negative and depressive symptoms. In earlier studies, the prevalence of antidepressant use in schizophrenia has been evaluated to be from 15% to 58%, however, there are differences between regions and how prevalence was measured^3–11^. Our previous study, which evaluated antidepressant use in patients with first-episode schizophrenia found that 35.4% initiated antidepressant use within 3 years after schizophrenia diagnosis^3^.

Only a few studies have assessed the effectiveness of antidepressant use in persons with schizophrenia in real-world settings. A previous U.S. study evaluated several outcomes related to the effectiveness and safety aspects of antidepressant augmentation^12^, and two previous studies have assessed the association between antidepressant use and mortality in schizophrenia in Nordic countries^9,13^, and one study in Taiwan^10^. To our knowledge, there are no previous studies investigating the effectiveness of specific antidepressants in real-world settings. The efficacy of antidepressant use in persons with schizophrenia has been investigated in several randomized controlled trials (RCTs) and meta-analyses based on these RCTs. Cochrane reviews conclude that antidepressants may be useful in improving negative symptoms^14^, but there is not enough evidence to support the treatment of depressive symptoms with antidepressants in schizophrenia^15^. Regarding add-on antidepressant treatment Helfer et al. included 82 studies with 3608 persons in their meta-analysis and concluded that add-on antidepressant treatment may have a small beneficial effect on negative and depressive symptoms and seem relatively safe to use^16^. Similar conclusions on antidepressant augmentation were derived in a meta-analysis by Galling et al. They concluded, based on 42 studies with a total of 1934 persons, that antidepressant augmentation to antipsychotics may be effective in reducing total and negative symptoms, although the finding on total symptoms may be driven by a reduction in negative symptoms^17^. These meta-analyses also noted several limitations, such as a small number of double-blinded trials, reporting bias, small study sample sizes, and confounding by secondary negative symptoms.

According to the Finnish Current Care Guideline^18^, antidepressants may be useful to treat negative and depressive symptoms in persons with schizophrenia. Different care guidelines seem to be relatively cautious^19–21^ with their guidance concerning the use of antidepressants in persons with schizophrenia, or state that their use is beyond the scope of the guideline^22^. Some even refrained from making recommendations due to the low quality of available evidence^23^. However, the Scottish guideline for the management of schizophrenia proposes that antidepressants may be trialed for persistent negative symptoms and depressive symptoms^24^.

The objectives of this study were to investigate the real-world effectiveness of antidepressant use in persons with schizophrenia, with psychosis hospitalization as the main outcome, and risk of hospitalization due to non-psychiatric and cardiovascular reasons, and all-cause mortality as secondary outcomes.

## Methods

### Study population

This cohort study included all persons treated in inpatient care due to schizophrenia in Finland (*N* = 61,889) during 1972–2014. Schizophrenia was defined as having an International Classification of Diseases (ICD) version 10 diagnosis of schizophrenia (F20) or schizoaffective disorder (F25) recorded in inpatient care. The data regarding diagnoses (including dates of admissions and discharges with discharge diagnoses) were obtained from the Hospital Discharge Register maintained by the National Institute for Health and Welfare. Prescription Register maintained by the Social Insurance Institution was utilized to obtain drug use information. Prescription Register data included ATC code, dispensing dates, purchased amount, and drug formulation (strength, package size, drug form). Data concerning dates of death and recorded causes of death were obtained from the National Death Register maintained by Statistics Finland. Data in the Prescription Register is available since 1995 and thus, follow-up for drug use in this study started at the beginning of 1996. Persons entered the cohort and the follow-up started at January 1996, or after the first diagnosis of schizophrenia for those diagnosed between 1996 and 2014. The follow-up ended either December 31, 2017 (end of data linkage) or at death.

### Exposure

The exposure of this study was the use of antidepressants (N06A), which were also categorized by mechanism of action (non-selective monoamine reuptake inhibitors (TCAs, N06AA), selective serotonin reuptake inhibitors (SSRIs, N06AB) and serotonin-norepinephrine reuptake inhibitors (SNRIs, including venlafaxine, milnacipran, and duloxetine). For the analyses of specific drug substances, nine most used antidepressants and antidepressant polytherapy (defined as time periods when two or more antidepressants were used concomitantly) were included. PRE2DUP (From Prescriptions to Drug Use Periods) method was utilized to model the data from drug dispensings to drug use periods. PRE2DUP estimates drug use periods on sliding averages of daily doses, purchased amount, and drug package-specific parameters, which control the joining of purchases. PRE2DUP also considers possible inpatient care, stockpiling of drugs, and dosage changes. PRE2DUP has been described in detail previously^25^ and the validity of the method has been shown to be good for antidepressants^26^. As register data do not include drugs used during inpatient stays time in hospital care is removed from all analyses (i.e. inpatient time is not categorized as “use” nor “non-use”).

### Outcomes

Main outcome was hospitalization due to psychosis (ICD-10: F20–F29). Secondary outcomes were non-psychiatric hospitalization (diagnoses other than F00–F99), hospitalization due to cardiovascular reasons (I00–I99), and all-cause mortality. Deaths recorded during the outpatient time were included, in addition to those happening during the first 2 days after admission to hospital care.

### Statistical analysis

Within-individual design was utilized to minimize selection bias in the analyses of hospital-based outcomes. In this design, each person acts as his/her own control, and only time-varying covariates were adjusted for, as the impact of all time-invariant factors is eliminated by the design. Time-varying covariates included were sequential order of treatments, time since cohort entry and use of antipsychotics (ATC N05A excl. lithium), mood stabilizers: carbamazepine (N03AF01), lamotrigine (N03AX09), lithium (N05AN01) and valproic acid (N03AG01), benzodiazepines (N05BA, N05CD) and Z-drugs (N05CF). Details of the covariates are presented in Supplementary Table 1. We used stratified Cox regression to calculate adjusted hazard ratios (aHR) with 95% confidence intervals (CI).

Mortality was investigated in the traditional between-individual Cox model with time-varying exposure and covariates. One-time events such as death cannot be analyzed in within-individual design and between-individual analysis was also conducted as a sensitivity analysis for the main outcome. Between-model was adjusted for the same covariates as within-individual analyses and additionally for age, sex, number of previous hospitalizations due to psychoses, asthma/chronic obstructive pulmonary disease, cardiovascular disease, cancer, diabetes, substance use disorder, previous suicidal behavior, type of schizophrenia-spectrum disorder (schizoaffective vs. schizophrenia), liver diseases, renal diseases, and medication use, including antiepileptics, statins, antidiabetics, anti-parkinson drugs and prior use of long-acting injectable antipsychotics, or clozapine. Covariates included in between-individual model are described in Supplementary Table 1.

Stratified analyses by sex were conducted for the main outcome. We also performed a sensitivity analysis for the main outcome by omitting the first 30 days of each exposure from analyses, since the effectiveness of antidepressant medications is rarely immediate. Data management and analyses were conducted with SAS 9.4.

Permissions for this study were granted by pertinent institutional authorities at the Finnish National Institute for Health and Welfare, The Social Insurance Institution of Finland, and Statistics Finland.

## Results

Altogether 61,889 persons were included in the study, and 50.3% were men (*N* = 31,104). The mean age at cohort entry was 46.2 [standard deviation, SD 16.0]. Characteristics of antidepressant users (49.3% of the cohort, *N* = 30,508) are described in Table 1. Number of events, number of users, and person-years per exposure are presented in Table 2. Most commonly used antidepressants were citalopram (used by 21.0% of the cohort), mirtazapine (12.6%), sertraline (8.3%), followed by fluoxetine (7.3%), and escitalopram (6.8%).

**Table 1:** Characteristics of antidepressant users (*N* = 30,508).

Characteristics/comorbidity	% (*N*)
Female gender	51.8 (15,815)
*Age categories at cohort entry*	
≤35 years	36.2 (11,033)
36-55 years	44.7 (13,646)
>55 years	19.1 (5829)
*Time since first diagnosis of schizophrenia at cohort entry*	
≤1 year	46.1 (14,056)
>1–5 years	10.2 (3111)
>5 years	43.7 (13,341)
*Number of previous hospitalizations due to psychoses at cohort entry*	
1	32.4 (9870)
2-3	30.9 (9414)
>3	36.8 (11,224)
*Comorbidities at cohort entry*	
Cardiovascular diseases	13.8 (4215)
Diabetes	6.4 (1948)
Cancer	2.4 (717)
Asthma/chronic obstructive disease	3.0 (916)
Substance use disorder	18.2 (5556)
Previous suicide attempt	12.6 (3853)
*Comorbidities diagnosed by the end of the follow-up*	
Cardiovascular diseases	35.7 (10,904)
Diabetes	17.4 (5298)
Cancer	11.3 (3454)
Asthma/chronic obstructive disease	10.2 (3122)
Substance use disorder	25.7 (7845)
Previous suicide attempt	18.2 (5557)

Cohort entry refers to entry into the schizophrenia cohort.

**Table 2:** Number of antidepressant users, events (hospitalizations due to psychosis), and person-years per exposure among antidepressant users.

	Users (N)	Events	Person-years
Any antidepressant use	30,508	44,522	181,638
Citalopram	13,020	13,322	56,188
Mirtazapine	7823	4140	16,911
Sertraline	5153	4198	17,390
Fluoxetine	4524	3623	14,879
Escitalopram	4237	2568	9826
Venlafaxine	4128	3766	13,416
Amitriptyline	2855	1568	7984
Paroxetine	2097	1933	6976
Mianserin	1812	1138	5104
Polytherapy	9553	3979	13,938
Other antidepressants	7023	4287	19,028
Non-use of antidepressants	59,687	131,657	642,242

**Fig. 1:**
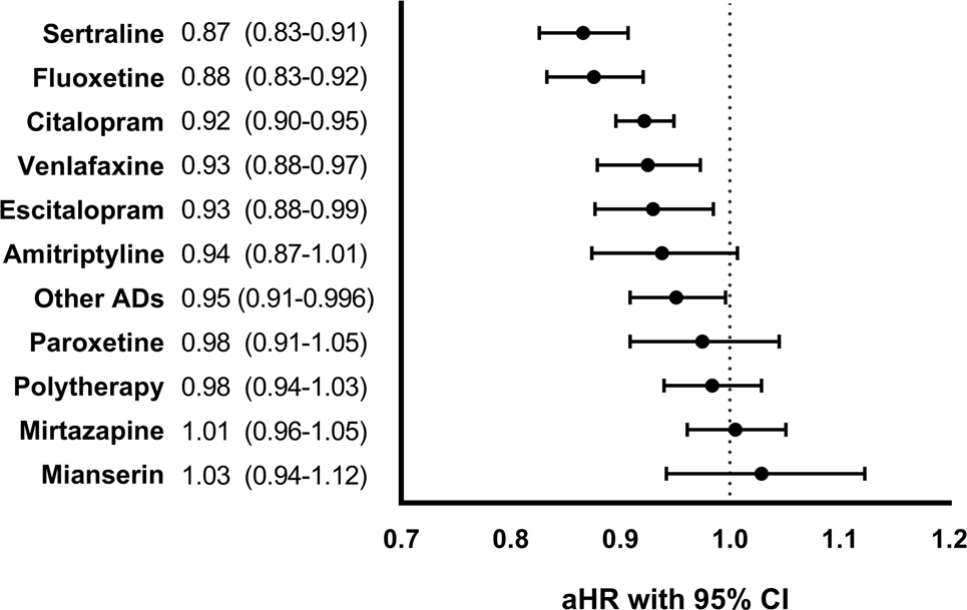
Risk of hospitalization due to psychosis associated with antidepressant (AD) use compared to non-use of antidepressants in within-individual design. Adjusted hazard ratios (aHRs) with 95% Confidence Intervals (CIs). Polytherapy refers to the concomitant use of two or more antidepressants. Other ADs include the rest of the antidepressants marketed in Finland.

During the follow-up (median 14.8 years, IQR 7.5–22.0), 36,923 (59.7%) persons were rehospitalized due to psychosis at least once. Antidepressant use was associated with a lower risk of psychosis hospitalization compared to non-use (aHR 0.93, 95% CI 0.92–0.95). Similar lower risks were observed within antidepressant groups SSRIs (aHR 0.91, 95% CI 0.89–0.93), SNRIs (aHR 0.92, 95% CI 0.88–0.97), and TCAs (aHR 0.93, 95% CI 0.89–0.98). Of the individual drug substances, use of sertraline (aHR 0.87, 95% CI 0.83–0.91), fluoxetine (0.88, 0.83–0.92), citalopram (0.92, 0.90–0.95), venlafaxine (0.93, 0.88–0.97) and escitalopram (0.93, 0.88–0.99) and other antidepressants (0.95, 0.91–0.996) was associated with lower risk of psychosis hospitalization (Fig. 1).

In sensitivity analyses, we observed similar results as in the main analysis when comparing men and women (Supplementary Fig. 1). We also performed an analysis of the main outcome when the first 30 days were omitted from all use and non-use periods, and the results were similar as in the main analysis (Supplementary Fig. 2). In a between-individual analysis of the main outcome, the results were generally similar, except for antidepressant polytherapy, which was associated with an increased risk of psychosis hospitalization (aHR 1.09, 95% CI 1.02–1.17) (Supplementary Fig. 3).

**Fig. 2:**
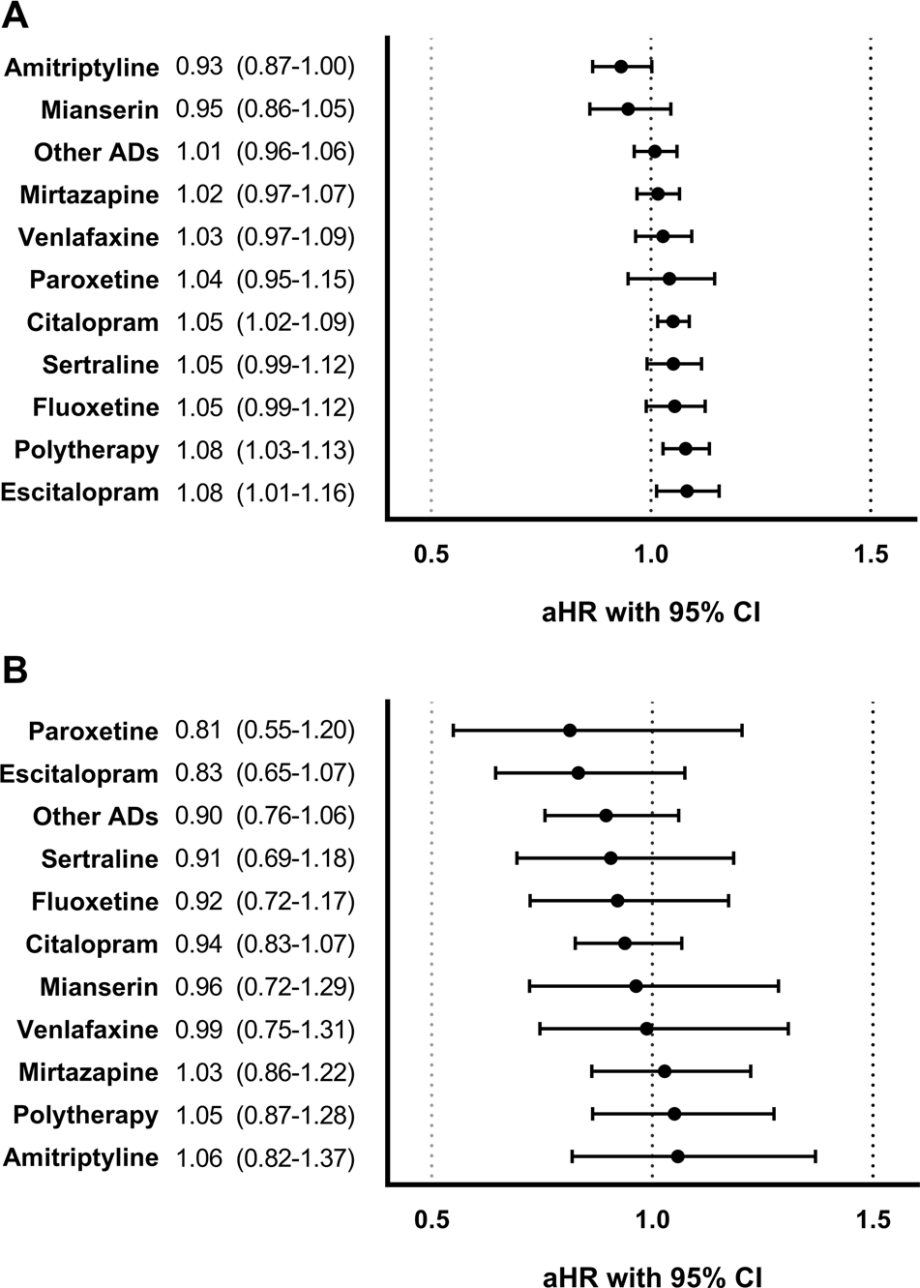
Risk of hospitalization associated with antidepressant (AD) use compared to non-use of antidepressants in within-individual design. Hospitalization due to **A** non-psychiatric reasons and **B** cardiovascular reasons. Adjusted hazard ratios (aHRs) with 95% Confidence Intervals (CIs). Polytherapy refers to the concomitant use of two or more antidepressants. Other ADs include the rest of the antidepressants marketed in Finland.

During the follow-up, 43,609 (70.5%) persons were hospitalized due to non-psychiatric reasons at least once, with 12,696 (20.5%) persons being hospitalized due to cardiovascular reasons. We found an increased risk of non-psychiatric hospitalization associated with antidepressant use compared to non-use (aHR 1.03, 95% CI 1.01–1.06). Considering individual drugs, this increased risk was observed with citalopram (aHR 1.05, 95% CI 1.02–1.09), escitalopram (aHR 1.08, 95% CI 1.01–1.16), and polytherapy (aHR 1.08, 95% CI 1.03–1.13) (Fig. 2A). We found no association with risk of hospitalization due to cardiovascular reasons during any use (aHR 0.95, 95% CI 0.87–1.03) compared to non-use of antidepressants (Fig. 2B).

**Fig. 3:**
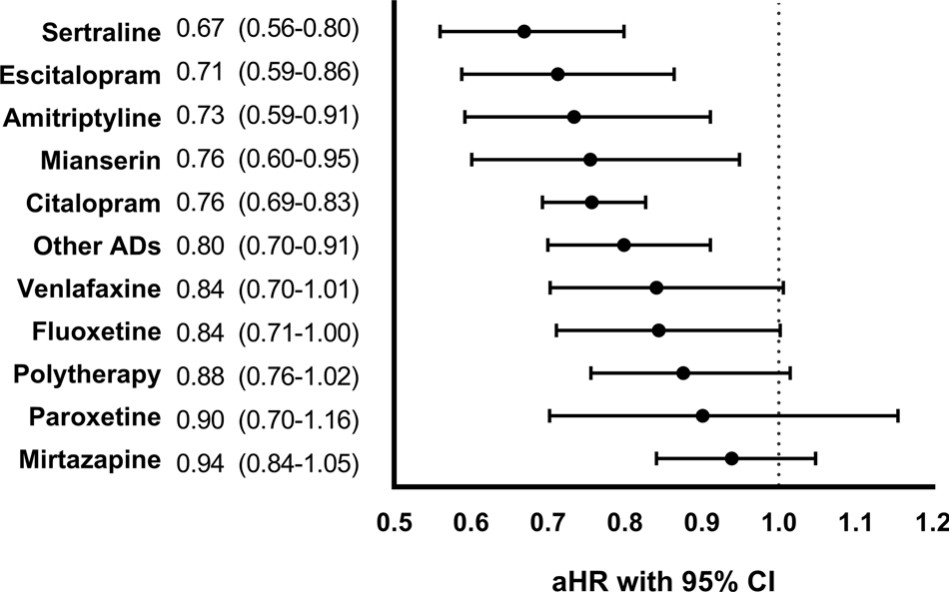
Risk of all-cause mortality associated with antidepressant (AD) use compared to non-use of antidepressants in between-individual design. Adjusted hazard ratios (aHRs) with 95% Confidence Intervals (CIs). Polytherapy refers to the concomitant use of two or more antidepressants. Other ADs include the rest of the antidepressants marketed in Finland.

Altogether 14,813 persons died during the follow-up. The risk of all-cause mortality was lower with antidepressant use compared to non-use (aHR 0.80, 95% CI 0.76–0.85). This lower risk was observed with sertraline (aHR 0.67, 95% CI 0.56–0.80), escitalopram (0.71, 0.59–0.86), amitriptyline (0.73, 0.59–0.91), mianserin (0.76, 0.60–0.95), citalopram (0.76, 0.69–0.83) and other antidepressants (0.80, 0.70–0.91) compared with non-use of antidepressants (Fig. 3).

## Discussion

In this study, we found that antidepressant use was associated with about 10% decreased risk of hospitalization due to psychosis and 20% decreased risk of all-cause mortality. None of the studied drugs were related to an increased risk of psychosis or mortality. However, we found that the use of antidepressants was associated with a slightly increased risk of hospitalization due to non-psychiatric reasons.

We found an association between a lower risk of psychosis hospitalization with antidepressant use in general and with certain antidepressants. This is in line with the findings of Stroup et al. who found a lower risk of hospital admission due to psychiatric reasons for antidepressant initiators (aHR 0.84, 95% CI 0.80–0.88)^12^. However, they investigated add-on antidepressant use comparing it with the initiation of another antipsychotic as a reference, and included hospitalization due to any mental disorder for this outcome. These differences make direct comparisons between the studies difficult. Stroup and colleagues did not assess specific antidepressants for this outcome. Our study showed a lower risk of psychosis hospitalization associated with sertraline, fluoxetine, citalopram, venlafaxine, and escitalopram. We did not observe a lower risk associated with mirtazapine and mianserin use. One reason for this might be that they are widely used for insomnia in Finland, and usually, the dosage with this indication is relatively small compared to other indications, especially with mirtazapine^27^. We found an association with antidepressant polytherapy, referring to the concomitant use of two or more antidepressants, and increased risk of psychosis hospitalization in between-individual analysis. More severely ill patients are often treated with several medications, which might explain this finding.

We found a 20% lower risk of mortality associated with antidepressant use. Similar findings considering antidepressant use have been found in a previous study comparing different levels of antidepressant exposure with non-use (15–40% lower risk depending on the level of exposure)^9^. Another study comparing the levels of antidepressant exposure found 17% lower risk associated with low exposure and 9% lower risk with moderate exposure, but there was no difference in risk with the highest exposure^10^. Similar trend was also seen in a previous Finnish study, even though the results were not statistically significant (HR 0.57, 95% CI, 0.28–1.16)^13^. However, the study cohort was considerably smaller (*N* = 2588) than in our study. Stroup and colleagues also evaluated mortality outcomes and they did not find an association between antidepressant use and mortality (aHR 0.97, 95% CI 0.81–1.17)^12^. Considering specific drugs, we found a lower risk of mortality with sertraline, escitalopram, amitriptyline, mianserin, and citalopram compared to non-use of antidepressants, and none of the studied specific drugs was associated with increased risk of mortality.

There was a somewhat increased risk of non-psychiatric hospitalization associated with citalopram (5% increased), escitalopram (8%) use, and polytherapy (8%). Citalopram and escitalopram were so widely used altogether that this may have affected to results of all antidepressants observed as a whole. Similarly, as Stroup and colleagues^12^, we found no association with cardiovascular hospitalizations, and it seems we lacked the statistical power with this outcome (as indicated by wide confidence intervals). Antidepressant use has been associated with certain adverse events^28^, such as fractures, cardiovascular events, and hyponatremia. However, if these adverse effects would be very severe, contrary to what we found, it probably would have been seen as a higher risk of mortality. Another possible explanation for the small increase in the risk of non-psychiatric hospitalization is that reduction of negative or depressive symptoms, or reduction in diagnostic overshadowing led to improvement in detection of physical diseases and consequently, resulted in a necessary hospitalization.

The strengths of this study are that we used a large nationwide study cohort, including all persons diagnosed with schizophrenia in inpatient care in Finland during a moderately long time period. The inpatient care register has high specificity to identify schizophrenia diagnosis (i.e. no false positive cases)^29^. In addition, our study enabled a long-term follow-up of up to 22 years, which is required for relatively rare outcomes such as mortality. PRE2DUP method, which we used in this study to derive time periods when drugs were used vs. not used, has been evaluated to estimate antidepressant and antipsychotic use well^26,30^. For the hospitalization-based outcomes, we used within-individual design, which eliminates the impact of all time-invariant factors such as sex and needs to be adjusted for only time-varying factors. These were sequential order of treatments, time since cohort entry, and use of certain medications.

There are certain limitations in this study. We did not have information on depressive symptoms which can act as a confounder. It is likely that antidepressant use is initiated when the symptoms are present or worsening but the full efficacy of antidepressants is reached after a delay of 2–4 weeks. To overcome this, we ran analyses by omitting the first 30 days from all exposures and the results remained nearly the same as in the main analysis. However, as it was not possible to remove the impact of fluctuation or severity of depressive symptoms in time, our results on the effectiveness of antidepressants in preventing hospitalizations due to psychosis might be an underestimation. Although the diagnosis of schizophrenia is often made in inpatient care, some patients with a less severe course of illness who have been treated only in outpatient care may be missing from our study cohort. Adherence to medication is a common problem in persons with schizophrenia. From the register-based data, we only have information on the dispensed drugs, and it is possible that they are not actually taken. Another limitation is that Finnish registers do not record indications for prescribed medications, i.e., mirtazapine might have been used for the treatment of insomnia, often with relatively low doses, which might affect our results.

In conclusion, antidepressant use was associated with decreased risk of hospitalization due to psychosis and lower mortality among patients with schizophrenia. Although there was a somewhat increased risk of non-psychiatric hospitalization associated with citalopram and escitalopram, we observed a decreased risk of mortality with these substances and with antidepressant use in general, which suggests that antidepressants are relatively safe to use in persons with schizophrenia. However, more studies evaluating the safety and benefits of antidepressant use in persons with schizophrenia are needed.

## Supplementary information

Supplementary information

## Data Availability

Study datasets include health data and therefore are not publicly available to secure participant privacy. Researchers can apply access to the data from the pertinent register holders: the Social Insurance Institution of Finland (Prescription Register), the National Institute for Health and Welfare (Hospital Discharge Register), and Statistics Finland (National Death Register).
