# Supplementary material for: Real-world effectiveness of antidepressant use in persons with schizophrenia: within-individual study of 61,889 subjects
Source: Schizophrenia (Heidelb). 2023 May 26;9(1):34. doi: 10.1038/s41537-023-00364-x (PMC10220047; doi:10.1038/s41537-023-00364-x)

**SUPPLEMENTARY MATERIAL:**

**Puranen A, Koponen M, Lähteenvuo M, Tanskanen A, Tiihonen J, Taipale H: Real-world effectiveness of antidepressant use in persons with schizophrenia: within-individual study of 61,889 subjects**

**Supplementary Table 1.** Definition of covariates

| **Definition** | **ICD-code/ATC-code/ categorization** | **Measurement period** | **Data source** |
| --- | --- | --- | --- |
| **Covariates in within-individual analysis** | | | |
| Sequential order of treatments | For antidepressants (N06A) | During the follow-up | PR |
| Time since cohort entry | Categorized as ≤1, 1-3, >3 years | Time-dependently during follow-up |  |
| Antipsychotics | N05A excluding Lithium (N05AN01) | Time-dependently during follow-up | PR |
| Mood stabilizers | Carbamazepine (N03AF01), Valproic acid (N03AG01), Lamotrigine (N03AX09), Lithium (N05AN01) | Time-dependently during follow-up | PR |
| Benzodiazepines | N05BA, N05CD | Time-dependently during follow-up | PR |
| Z-drugs | N05CF | Time-dependently during follow-up | PR |
|  |  |  |  |
| **Covariates in between-individual analysis (in addition to covariates in within-individual analysis)** | | | |
| Age, years | Categorized as  ≤35, 36-55, >55 years | At cohort entry | HDR |
| Gender | Male or female |  | HDR |
| Number of previous hospitalizations for psychosis | Categorized as: 1, 2-3, 4-5, 6-9, ≥10  Defined as hospitalization with main diagnosis of:  ICD-10: F20-F29;  ICD-9: 295, 297, 298, 3010, 3012;  ICD-8: 295, 297, 298, 29999, 3010, 3012 | Since 1972 until cohort entry | HDR |
| Asthma/ COPD | ICD-10: J44-J46;  ICD-9: 493, 496;  ICD-8: 493 | Measured during 1972-2017, coded as “no” until the date of first diagnosis and “yes” there after | HDR |
| Cardiovascular disease | ICD-10: I00–I99;  ICD-9: 40-45;  ICD-8: 40-45 | Measured during 1972-2017, coded as “no” until the date of first diagnosis and “yes” there after | HDR |
| Cancer | ICD-10: C00–C97;  ICD-9: 140-208;  ICD-8: 140-208 | Measured during 1972-2017, coded as “no” until the date of first diagnosis and “yes” there after | HDR |
| Diabetes | ICD-10: E10-E14;  ICD-9: 250;  ICD-8: 250;  or medication use (ATC-code A10) | Measured during 1972-2017, coded as “no” until the date of first diagnosis or first drug dispensing and “yes” there after | HDR, PR |
| Substance use disorder | Hospitalization  (ICD-10: F10-F19, K86.0, K70;  ICD-9: 291, 292, 2940A, 303, 304, 305, 5770D-F, 5771C, 5771D, 5710A, 5711A, 5712A, 5713X;  ICD-8: 291, 303, 304, 57700-57708);  or medication use (ATC-codes N07BB, N07BC01, N07BC02, N07BC51) | Measured during 1972-2017, coded as “no” until the date of first diagnosis or first drug dispensing and “yes” there after | HDR, PR |
| Previous suicidal behavior | ICD-10: X60–X84, Y10–Y34, Z72.8, Z91.5;  ICD-9: E950A, E954A, E955A, E956A, E957A, E959A, E959X, E970A, E972A, E973A, E974A, E979A;  ICD-8: E950-E959 | Measured during 1972-2017, coded as “no” until the date of first diagnosis and “yes” there after | HDR |
| Type of SSD | ICD-10: schizophrenia (F20), schizoaffective disorder (F25) based on most recent diagnosis | At cohort entry | HDR |
| Liver disease | ICD-10: K70-K77 | Measured during 1996-2017, coded as “no” until the date of first diagnosis and “yes” there after | HDR |
| Renal disease | ICD-10: N10-N19 | Measured during 1996-2017, coded as “no” until the date of first diagnosis and “yes” there after | HDR |
| **Medications** | | | |
| Antiepileptics | N03AA02, N03AA03, N03AB02, N03AB52, N03AD01, N03AE01, N03AF02, N03AF04, N03AG04, N03AG06,  N03AX excluding  lamotrigine (N03AX09) | Time-dependently during follow-up | PR |
| Statins | C10A, C10B | Time-dependently during follow-up | PR |
| Antidiabetics | A10A, A10B | Time-dependently during follow-up | PR |
| Antiparkinsonian | N04AA | Time-dependently during follow-up | PR |
| Prior LAI | Long-acting injectable antipsychotics | LAI use before cohort entry | PR |
| Prior use of clozapine | N05AH02 | Before cohort entry | PR |
| ATC, Anatomical Therapeutic Chemical; COPD, chronic obstructive pulmonary disease; HDR, Hospital Discharge Register; ICD, International Classification of Diseases; PR, Prescription Register; SSD, schizophrenia-spectrum disorder; Z-drugs, benzodiazepine related drugs | | | |

**Supplementary Figure 1.** Risk of psychosis hospitalization associated with antidepressants compared with non-use of antidepressants in men and women in within-individual design. Adjusted Hazard Ratios (aHRs) with 95% Confidence Intervals (CIs). Polytherapy refers to concomitant use of two or more antidepressants. Other ADs include rest of the antidepressants marketed in Finland.


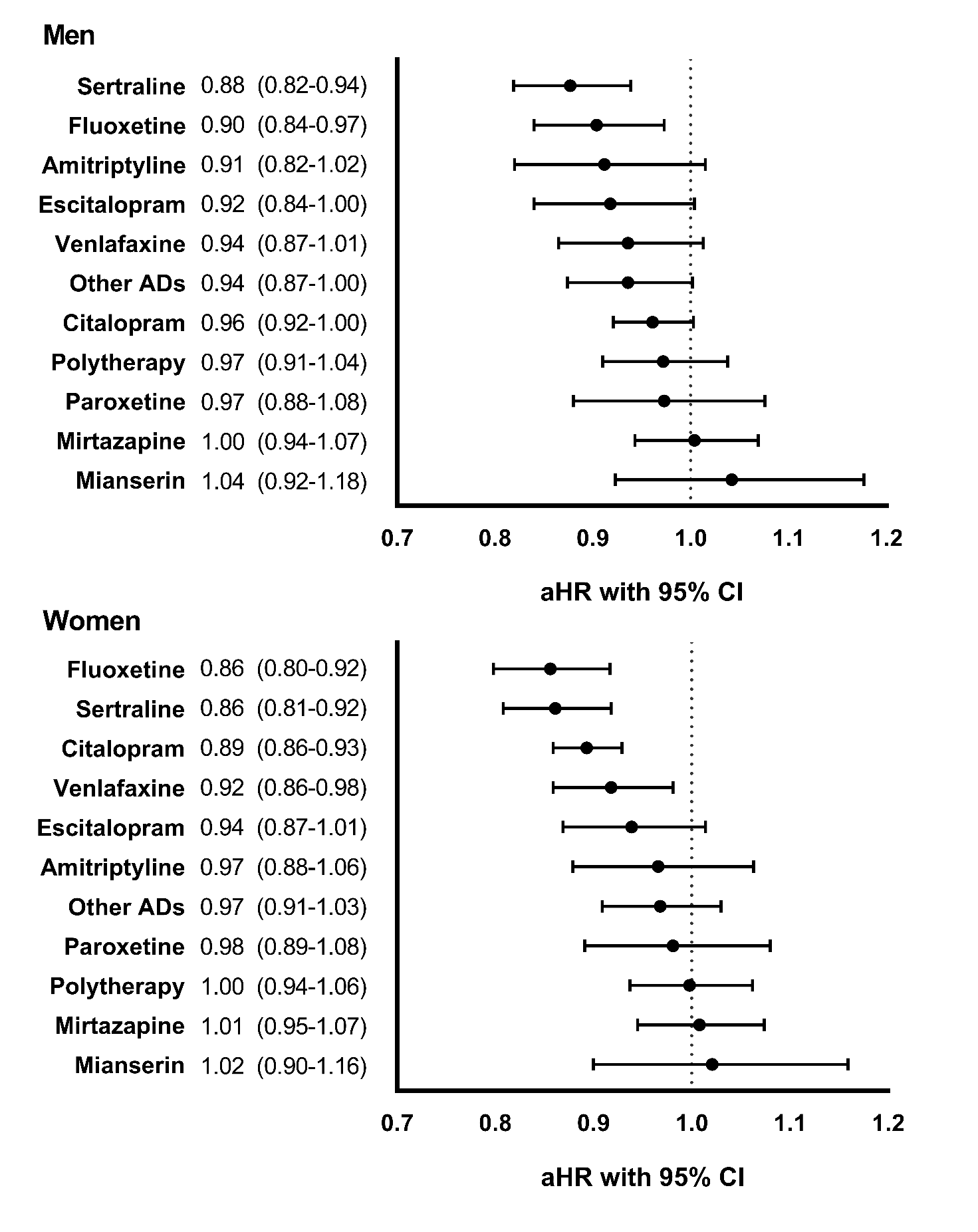


**Supplementary Figure 2.** Risk of psychosis hospitalization associated with antidepressants and compared with non-use of antidepressants in within-individual design with 30 first days of each exposure omitted. Adjusted Hazard Ratios (aHRs) with 95% Confidence Intervals (CIs). Polytherapy refers to concomitant use of two or more antidepressants. Other ADs include rest of the antidepressants marketed in Finland.


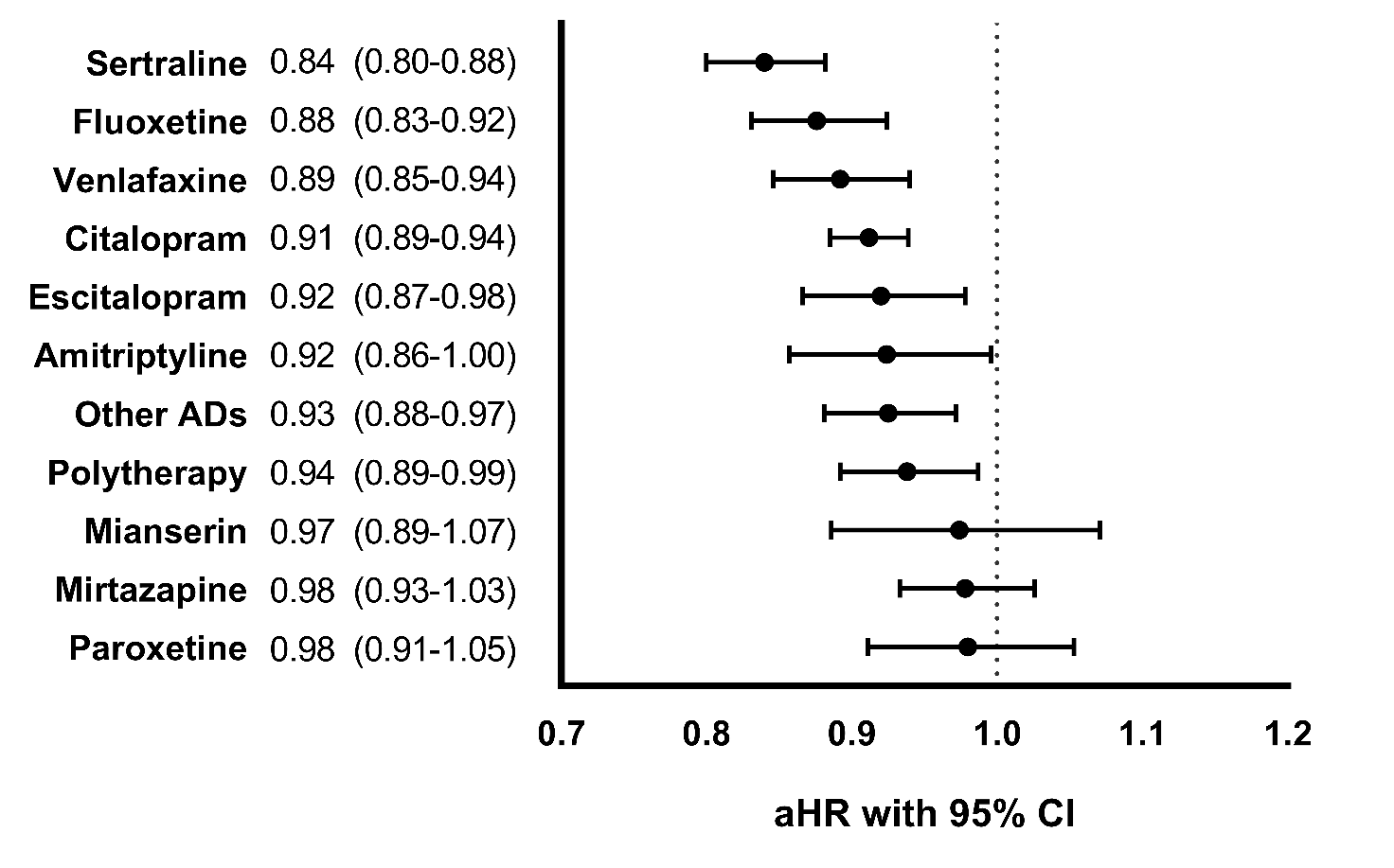


**Supplementary Figure 3.** Risk of psychosis hospitalization associated with antidepressants and compared with non-use of antidepressants in between-individual design. Adjusted Hazard Ratios (aHRs) with 95% Confidence Intervals (CIs). Polytherapy refers to concomitant use of two or more antidepressants. Other ADs include rest of the antidepressants marketed in Finland.


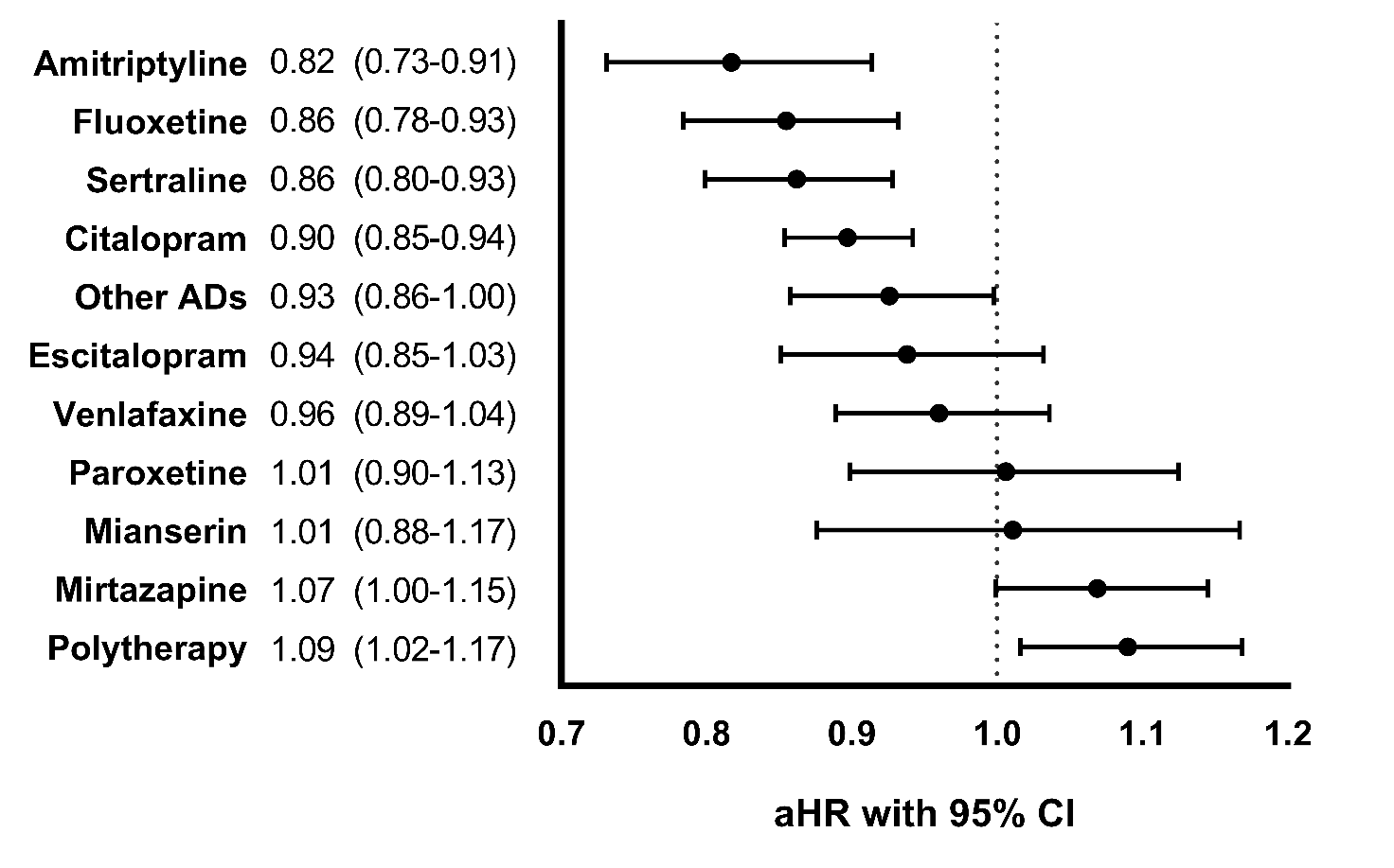

Supplement: Supplementary file 1 — Supplementary information [file 41537_2023_364_MOESM1_ESM.docx]
